# Traumatic brain injury induces DNA damage in Drosophila

**DOI:** 10.17912/micropub.biology.001756

**Published:** 2025-10-21

**Authors:** Rebeccah J Katzenberger, Barry Ganetzky, David A Wassarman

**Affiliations:** 1 Department of Medical Genetics, University of Wisconsin-Madison; 2 Department of Genetics, University of Wisconsin-Madison

## Abstract

Traumatic brain injury (TBI) is a major public health concern, affecting millions of people worldwide each year. Older individuals who experience a TBI face a higher risk of cognitive decline, disability, and mortality compared with younger individuals. A well-documented molecular consequence of TBI in both humans and rodent models is DNA damage. We used a *Drosophila melanogaster* (fruit fly) TBI model to investigate when DNA damage occurs following injury and whether age at the time of injury affects its severity. Using a Comet assay, which quantifies DNA damage in individual cells, we found that damage in the brain occurred within 4 hours of injury in both young and older flies. Levels of damage remained stable in young flies at 6 hours post-injury, but increased in older flies, indicating that aging processes enhance the post-TBI DNA damage mechanism. Although DNA damage initially resolved within 24 hours of injury; likely through DNA repair, loss of damaged cells, or death of flies with damage; it reappeared weeks later, revealing a previously unrecognized second phase of genomic instability following TBI. These findings establish Drosophila as a valuable model for studying TBI-induced DNA damage, a model that offers powerful genetic tools to investigate underlying mechanisms and to test whether genetic background affects the severity of DNA damage and contributes to individual variation in TBI outcomes.

**Figure 1. TBI induces DNA damage in the Drosophila brain:**
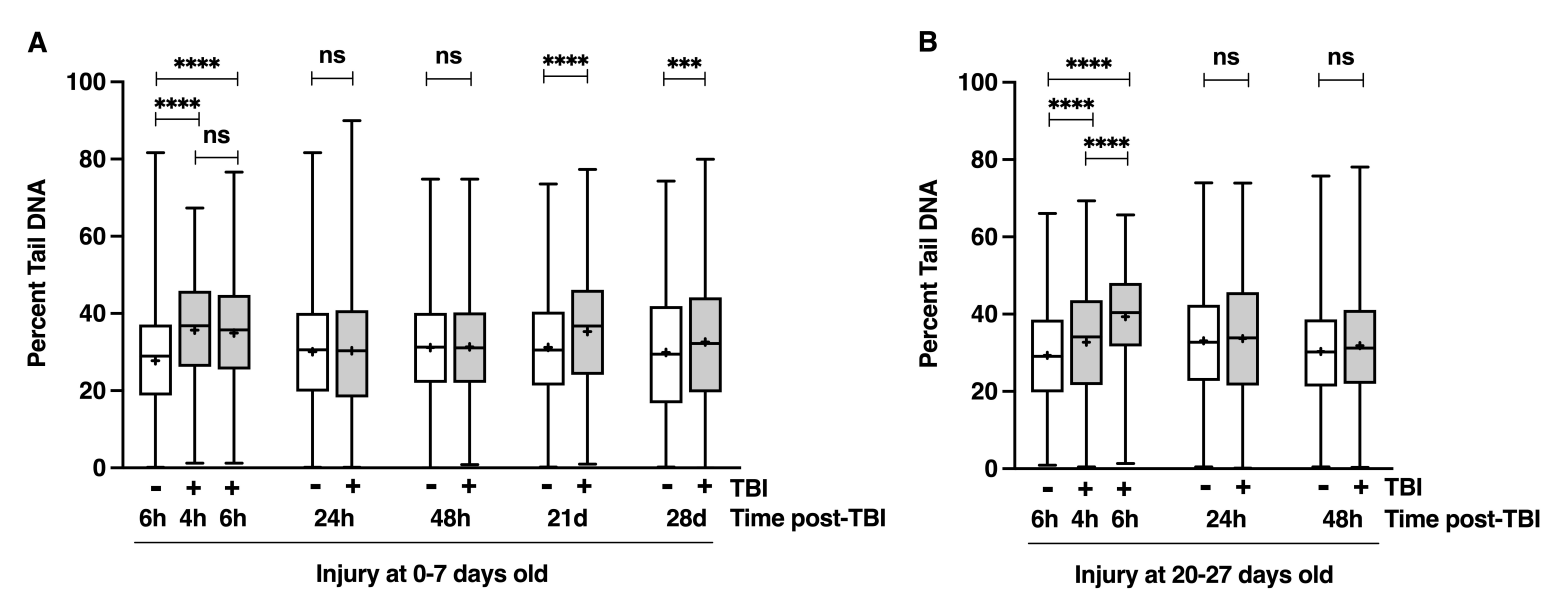
The Comet assay was used to determine the percent tail DNA in brain cells of male *w ^1118^* flies under the indicated injury and time post-injury conditions. (A) Flies injured at 0-7 days old and (B) flies injured at 20-27 days old. Percent tail DNA is shown as box-and-whisker plots, with the median (horizonal line), average (+), middle 50% of the data (box), and minimum and maximum values (whiskers). p-values reflect comparisons between two conditions using one-way analysis of variance (ANOVA) with Tukey’s multiple comparison test. ns=not significant, ***p<0.001, ****p<0.0001.

## Description

Traumatic brain injury (TBI) occurs when a mechanical force – such as a blow to the head – damages the brain. These forces can disrupt normal brain function, leading to temporary and long-term physical, cognitive, and emotional impairments (Blennow et al. 2012). With an estimated 70 million cases worldwide each year, TBI is a major public health concern (Taylor et al. 2017). Despite TBI’s prevalence, the cellular and molecular mechanisms underlying its outcomes remain incompletely understood, complicating efforts to develop effective preventive strategies and treatments.

DNA damage is a contributing factor to TBI pathology (Davis and Vemuganti 2021). Studies of rodent models of TBI have shown that following injury, affected brain cells accumulate various forms of DNA damage, including single- and double-strand breaks, chemical modifications of bases, and formation of R-loops – structures consisting of an RNA-DNA hybrid (Clark et al. 2001; Itoh et al. 2013; Baky et al. 2016; Davis et al. 2022; Schwab et al. 2022; Baral et al. 2025; Zhao et al. 2025). If unrepaired, these lesions can impair transcription, disrupt cell cycle regulation, and trigger inflammation and cell death, thereby contributing to both acute and chronic consequences of TBI (Zhao et al. 2023).

As part of our characterization of a Drosophila melanogaster (fruit fly) model of TBI, we investigated whether injury induces DNA damage. We inflicted TBI in flies using a High-Impact Trauma (HIT) device that consists of a metal spring anchored to a board, with the free end holding a plastic vial containing flies (Katzenberger et al. 2013). When the spring is pulled back and released, the vial rapidly strikes a polyurethane pad, delivering mechanical forces to flies through rapid deceleration as they collide with the vial wall. The HIT device produces both contact and inertial forces to the head and brain, along with concurrent forces to the body – thus modeling polytraumatic injury characteristic of most human TBIs, such as those from falls, vehicular accidents, and blast exposures (Yue et al. 2020).

We used the gel electrophoresis-based Comet assay to measure DNA damage in individual brain cells following TBI (Olive and Banáth 2006). In this assay, cells embedded in agarose are lysed and subjected to electrophoresis. Undamaged chromosomes remain largely immobile due to their large size, forming a ‘comet head.’ In contrast, fragmented DNA migrates toward the anode, forming a ‘comet tail.’ The percent of total DNA in the tail provides a quantitative measure of DNA damage. Since electrophoresis is performed under alkaline conditions, the assay detects single- and double-strand DNA breaks as well as alkali-labile sites in DNA such as abasic sites and oxidized sugar residues.

Male *w ^1118^* flies (a standard laboratory fly line) injured at 0-7 days old showed increased DNA damage in the brain at 4 and 6 hours post-injury compared to uninjured controls ( Fig. 1A ). DNA damage levels returned to baseline at 24 and 48 hours, but were increased again at 21 and 28 days after injury. Flies injured at 20-27 days old showed a similar pattern through 48 hours, but with greater damage at 6 hours than 4 hours ( Fig. 1B ). These findings show that TBI causes immediate DNA damage in both young and older fly brains; that this damage is repaired or eliminated through cell or fly death within 24 hours; that aging worsens the extent of damage; and that TBI also triggers a delayed, secondary wave of DNA damage.

Since these findings align with known effects in mammalian TBI models, they further validate Drosophila as a model for studying human TBI. The genetic tractability of flies can be exploited to identify genes and pathways that contribute to TBI-induced DNA damage. While it remains unclear whether the observed DNA damage results from oxidative stress due to mitochondrial dysfunction or from extensive fragmentation during late-stage apoptosis, either possibility warrants further investigation (Davis and Vemuganti 2021). The mechanism driving the secondary phase of DNA damage also remains unknown. It was observed at 21 days post-injury (i.e., in 21-28 day old flies) ( Fig. 1A ), but our findings do not reveal when this second phase begins. Interestingly, flies injured at 0-7 days old and surviving the initial 24 hours show normal survival for approximately 17 days but begin dying at a higher rate than uninjured flies at 17-24 days of age (Katzenberger et al. 2013), raising the possibility that the onset of late mortality is associated with the second wave of DNA damage.

## Methods

***Fly culturing and TBI***

Flies were maintained at 25°C on a standard type of fly food containing cornmeal, molasses, and yeast (Blommer et al. 2021). The *w ^1118^* strain has been maintained in our lab for many years. Flies were aged as indicated and subjected to TBI in groups of 60 using a HIT device (4 strikes, spaced 5 min apart), following the protocol described in Katzenberger et al. 2013.

***Comet assay***

Comet assays were performed and analyzed as described in Rimkus and Wassarman 2018. In brief, a Comet Assay Kit (Trevigen, Catalog #4250-050K) was used to analyze a minimum of 8 brains per time point, at least 4 biological replicates of 2 brains. Comets were imaged using a Zeiss LSM 510 confocal microscope with an Imager.M1 module using a 20X/0.8 NA Plan APOCHROMAT objective. For each condition, >225 comets were imaged using CometScore Pro Software (TriTek Corporation). GraphPad Prism (version 10.5.0) was used to generate graphs and perform statistical analysis using one-way ANOVA with Tukey’s post-hoc test to compare percent tail DNA between sample pairs.
